# A Comparison Study of Functional Groups (Amine vs. Thiol) for Immobilizing AuNPs on Zeolite Surface

**DOI:** 10.3390/nano9071034

**Published:** 2019-07-19

**Authors:** Xi Rao, Michaël Tatoulian, Cédric Guyon, Stephanie Ognier, Chenglin Chu, Ali Abou Hassan

**Affiliations:** 1 Key Laboratory of Luminescent and Real-Time Analytical Chemistry (Southwest University), Ministry of Education, School of Materials and Energy, Southwest University, Chongqing 400715, China; 2 Chimie ParisTech, PSL University, CNRS, Institut de Recherche de Chimie Paris, 75005 Paris, France; 3 Chongqing Key Laboratory of Soft-Matter Material Chemistry and Function Manufacturing, Southwest University, Chongqing 400715, China; 4 Jiangsu Key Laboratory for Advanced Metallic Materials, School of Materials Science and Engineering, Southeast University, Nanjing 211189, China; 5 Centre National de la Recherche Scientifique CNRS, Sorbonne Université, Physico-chimie des Electrolytes et Nanosystèmes InterfaciauX, Phenix, F-75005 Paris, France

**Keywords:** AuNPs, zeolite, immobilization, functional group

## Abstract

Immobilization of gold nanoparticles (AuNPs) on the surface of zeolite has received a great interest due to Au@zeolite’s unique characteristics and high performance for catalysis. In this work we studied the grafting of two different functional molecules; one having an amine group (3-aminopropyl)triethoxysilane (APTES) and the second having a thiol group (3-mercaptopropyl)trimethoxysilane (MPTES) on the surface of zeolite using the same wet chemistry method. The modified zeolite surfaces were characterized using zeta potential measurements; diffuse reflectance infrared fourier transform (DRIFT) and X-ray photoelectron spectroscopy (XPS). The results confirmed a successful deposition of both functional groups at the topmost surface of the zeolite. Furthermore; transmission electron microscopy (TEM), ultraviolet-visible (UV-Vis) spectroscopy and XPS results clearly evidenced that APTES provided a better AuNPs immobilization than MPTES as a result of; (1) less active functions obtained after MPTES deposition, and (2) the better attaching ability of thiol to the gold surface.

## 1. Introduction

Gold nanoparticles (AuNPs) have been considered as a kind of high performance catalyst in the last decades [1,2,3,4,5]. Although gold particles with a nano size are very attractive for catalysis, their aggregation can lead to a decrease in their catalytic activity [6,7]. For instance, the serious aggregation could strongly decrease the performance of gold to a very low level, even compared to group VIII metals [8]. In order to maintain a high activity of gold catalysts, support materials that could provide a uniform dispersion to AuNPs become a feasible solution.

Supported gold has gained attractive interest since its huge benefits in improving gold catalytic efficiency were reported [8,9,10,11]. Initially, reducible metal oxides, such as TiO_2_, ZnO and Fe_2_O_3_ were preferably employed as supports for gold [12,13]. Thereafter, the attractiveness of silica materials as supports for gold catalysts increased following reports on high catalytic activity of Au/SiO_2_ in reactions [14,15]. Non-oxides, like activated carbon (AC), were also slightly used as supports due to their unique advantages compared to oxides [16].

As one of the support material candidates for immobilizing gold, zeolite was used as a highly efficient support by several research groups in recent years. Due to its rigid structure with a three-dimensional framework forming channels and/or cages with molecular dimension, zeolite could atomically disperse a gold catalyst with a high degree of uniformity, consequently keeping high catalytic performance for AuNPs [17,18]. The gold loaded on Y type zeolite was reported to performed high activity for CO-O_2_ reaction [19]. Alternatively, the acid sites on zeolite could modify the electronic structure of AuNPs [20,21], such a synergistic interaction between the dual-active-sites of the acid sites and AuNPs was found to be responsible for the excellent catalytic performance in both experimental studies and theoretical calculations [22,23]. For instance, the good catalytic activity for alcohol oxidation by using Au@zeolite catalysts was evidenced in Zhang’s work [24]. Moreover, zeolite was also confirmed as an active phase for some chemical reactions [25,26,27]. Thus, the immobilization of AuNPs on zeolite to form Au@zeolite catalyst with synergistic effect is promising for catalysis.

Beside their spatial arrangement, the nature of the interaction between the AuNPs and the surface of the support also determines the catalytic performance of the gold catalyst. The method of immobilizing AuNPs on a support, often starts with an intermediate layer that consists in depositing on a solid surface an organic molecule with a functional group, which is able to attach AuNPs by covalent or electrostatic interaction [28,29,30]. On one hand, these connection layers containing functional groups must be stable for a relative long duration; on the other hand, interaction of the nanoparticles with the functional groups on the surface should be strong enough, guaranteeing that the AuNPs anchor to the surface upon reactions. In years, many linkage reagents with various functional groups were utilized for gold immobilization [28,30]. By adjusting the pH to allow for their protonation, amine groups can interact with negatively charged gold nanoparticles through electrostatic interaction. (3-aminopropyl)triethoxysilane (APTES) has been deemed to be one of the most popular linkage reagents for AuNPs immobilization, as it could provide amine groups for immobilizing gold, while the three hydrolysable ethoxy groups ensure a robust anchoring of the silane to the surface [30]. Alternatively, another interesting functionality is based on thiol groups, which can also bind gold surfaces due to strong covalent bonding [28]. Similar to APTES, (3-mercaptopropyl) trimethoxysilane (MPTES) is an organosilane with three alkoxy groups that could react with the hydroxyl groups on the surface of substrate to the formation of Si–O bonds, leaving the terminal functional thiol groups available for immobilizing AuNPs. Both molecules are often used for the immobilization of AuNPs, however, there are no quantitative proofs that demonstrate which functionality is better for attaching AuNPs. However, it has been announced that a weak interaction originates from electrostatic bonding, whereas a strong interaction is obtained from covalent bonding [29].

In the present work, we aimed to bring some answers to this question. APTES and MPTES molecules were deposited on the surface of zeolite using the same wet chemistry process, and the efficiency of the two linkage reagents were investigated by a further immobilization step of AuNPs on the as-deposited surfaces. Zeta potential measurements, diffuse reflectance infrared Fourier transform (DRIFT) and X-ray photoelectron spectroscopy (XPS) were used to characterize the as-deposited APTES and MPTES layers. In order to find the better functional group to immobilize AuNPs in terms of coverage and dispersion of nanoparticles, different methods such as transmission electron microscopy (TEM), ultraviolet-visible spectroscopy and XPS were employed.

## 2. Experimental

### 2.1. Materials and Chemicals

Chemicals were used as received: zeolite (Y type, CBV400, Zeolyst International, Delfzijl, Netherlands), (3-aminopropyl) triethoxysilane (98%, VWR, Fontenay-sous-Bois, France), (3-mercaptopropyl) trimethoxysilane (95%, VWR, Fontenay-sous-Bois cedex, France), gold (III) chloride hydrate (HAuCl4 · xH2O; Mw = 339.79 g/mol; 99.999%, St. Quentin Fallavier, France ) and silver nitrate (99%, Sigma-Aldrich, St. Quentin Fallavier, France) and trisodium citrate (99%, Alfa Aesar, Kandel, Germany ). Millipore water was used in all experiments.

### 2.2. Preparation of AuNPs

Five mL of Gold (III) chloride hydrate aqueous solution (1 wt.%), 420 μL of silver nitrate aqueous solution (0.1 wt.%) and 1.5 mL of sodium citrate aqueous solution (10 wt.%) were premixed and injected into 45 mL of boiling water kept under reflux [31]. The mixture was then heated for 30 min, which changed the color of the suspension into dark purple. Thereafter, the mixture was heated for another 30 min to complete the synthesis (no more color variation was observed) and then the suspension was let to cool down to room temperature. The final pH of the suspension was measured to be 4.2.

### 2.3. Salinization of the Surface of the Y Zeolite

Y zeolite was calcined at 500 °C in an oven with air flow for 12 h before it was used for any experiments. Hundred mg of as-calcined zeolite with 100 μL of silane reagent (APTES or MPTES) were mixed in 30 mL of anhydrous dichloromethane. The silanization of the zeolite was carried out at room temperature for 24 h by magnetic stirring. Then, the APTES/MPTES deposited zeolite samples were washed three times by centrifugation (4000 rpm) with 40 mL anhydrous dichloromethane. The collected samples were dried in oven at 100 °C for a whole night.

### 2.4. Immobilization of the AuNPs on Silane Modified Zeolite

Fifty mg of APTES/MPTES modified zeolite nanoparticles (NPs) were dispersed in 20 mL of a phosphate buffer (pH = 4.2) under sonication, then 10 mL of the AuNPs were added under continuous stirring at room temperature for 12 h. The AuNPs immobilized on the zeolite NPs were separated by centrifugation (4000 rpm) for at least three times and washed again with 40 mL water. Then, the AuNPs immobilized samples were dried in oven at 100 °C overnight.

### 2.5. Characterizations

A Zetasizer Nano ZS (Malvern Panalytical, Malvern, UK) was used for zeta-potential measurement and a phosphate buffered solution (10 mM) with pH value of 4.2 was used for the test. A Tensor 27 spectrometer (Bruker, Ettlingen, Germany) was employed for collecting the diffused reflectance infrared Fourier transform (DRIFT) spectra, and the recording range was from 4000 to 400 cm^−1^. A spectrophotometer (Ocean optics, Winter Park, FL, USA) was applied for obtaining the UV-Vis spectra. A PHI 5600-ci XPS spectrometer (Physical Electronics, Cambridge, UK) was used for collecting XPS spectra. The anode used was a monochromatic Al (1486.6 eV) and a Mg K α (1253.6 eV) X-ray sources at 200 W, respectively. A 2000FX microscope (JEOL, Tokyo, Japan) equipped with an energy-dispersive X-ray spectroscopy (EDS) was employed for capturing morphology images and identifying the amount of AuNPs; the operating voltage was 200 kV.

## 3. Results and Discussion

Zeta-potential analysis was performed to study the effect of grafting of both molecules on the surface charge of the zeolite, as well as after the deposition of AuNPs. The final pH of the AuNPs suspension was 4.2, and at such pH the suspension showed a high colloidal stability. All zeta potential measurements were carried out at the same pH using a phosphate buffer solution with pH = 4.2. Moreover, the zeolite nanoparticles also showed a high colloidal stability at this pH. The high colloidal stability of both suspensions is important to allow for a homogeneous assembly between the Au and the zeolite NPs, and good dispersion of the Au catalyst. The results of the zeta potential measurements were summarized in Table 1. They indicated that the initial zeta potential of the zeolite before any modification was about −39.7 mV. Such a negative value showed that they could hardly anchor the AuNPs, since they were also negatively charged with a zeta potential of about −35 mV at the same pH. After the surface functionalization of the zeolite with the silanes, the zeta potential decreased. When APTES and MPTES were used, the value decreased to −36.4 and −12.2 mV, respectively. Such a decrease confirmed the effective deposition of functional groups on the surface of the zeolite. Following AuNPs immobilization to the silanes pre-deposited zeolite, the zeta-potential increased again to −40.2 and −44.2 mV. The increase in the zeta potential indicated a successful immobilization of AuNPs, as the surface of AuNPs is negatively charged due to the existence of citrate anions on gold surface.

**Figure 1:**
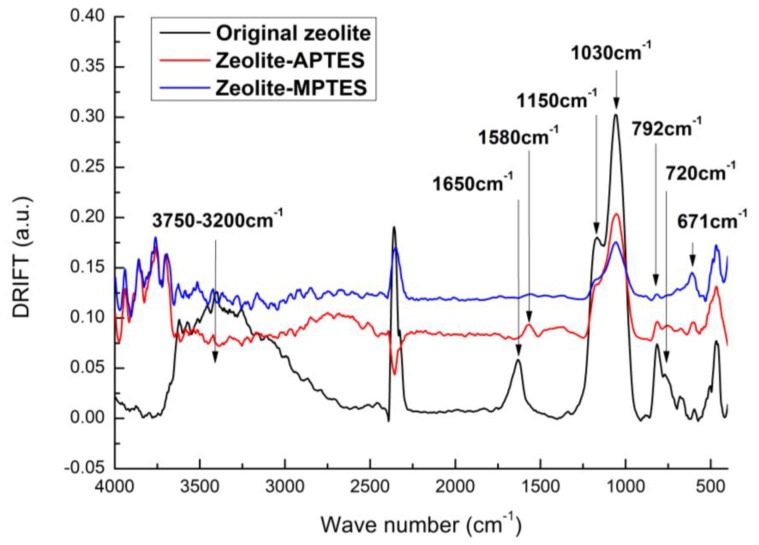
DRIFT spectra of zeolite before and after deposited with APTES or MPTES.

Furthermore, DRIFT was used to confirm the successful surface anchoring of the functional molecules on the surface of the zeolite. As shown in Figure 1, new absorption peaks could be observed after APTES and MPTES deposition. As known, zeolite is formed by T-O units (T = Si or Al frameworks) that have absorption features in DRIFT spectrum. The band at 1150 cm^−1^ was ascribed to the asymmetric stretching modes of the internal tetrahedral, while the band at 725 cm^−1^ was assigned to the symmetric stretching modes of the internal tetrahedral. The bands at 1030 and 792 cm^−1^ could respectively correspond to asymmetric and symmetric stretching modes of the external linkages [32]. Moreover, the broad peak around 3750~3000 cm^−1^ and the peak at 1650 cm^−1^ were ascribed to the −OH groups on the zeolite surface [33].

APTES deposited zeolite showed differences compared to the untreated zeolite. A new absorption peak at 1580 cm^−1^ was obviously seen, which could be assigned to the NH_2_ absorption feature. Besides, a decrease of the absorption peaks of the zeolite at 3750, 1650, 1150, 1030, 792 and 725 cm^−1^ were observed, implying a good coverage of APTES on zeolite. The spectrum of MPTES functionalized zeolite showed one absorption peak, representing the C-S stretch at 671 cm^−1^, with the same trend of decreasing original zeolite characteristic peaks also revealing the successful deposition of MPTES.

**Figure 2:**
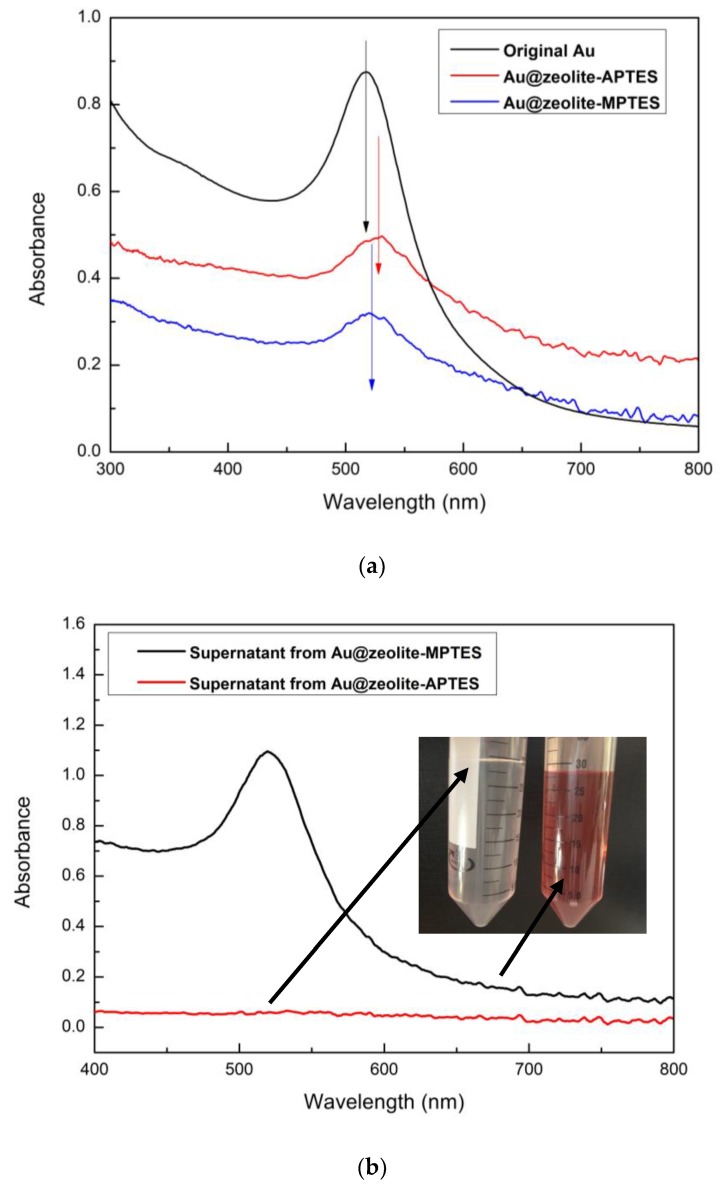
UV-Vis spectra of samples: (**a**) AuNPs immobilized zeolite; (**b**) supernatants collected after immobilization.

The immobilization of AuNPs by APTES and MPTES was also investigated using UV-Vis spectroscopy (Figure 2). Due to the surface plasmon resonance [34], the electron cloud can oscillate on the AuNPs surface, which leads to absorption of the visible light. Thus, an absorption peak at around 520 nm was observed from the spectrum of as-synthesized AuNPs colloid. Based on the literature [35], such SPR was coherent with an average diameter of the AuNPs of 10–15 nm. A similar peak was also observed in the spectra of the AuNPs immobilized zeolite, demonstrating that both APTES and MPTES are effective molecules for anchoring gold. The characteristic SPR peak shifted to ~528 nm in the spectrum of APTES deposited zeolite, while the SPR was ~523 nm in the spectrum of MPTES deposited zeolite. The larger red shift indicated a smaller distance between AuNPs on the attached surface. Thus, the results suggested a higher AuNPs density on the APTES deposited zeolite.

The supernatants were also collected and analyzed by UV-Vis to qualitatively check the attachment of AuNPs. After 12 h of contact of the AuNPs suspension with the APTES or MPTES modified zeolite, the Au@zeolite assemblies were isolated by centrifugation at 4000 rpm for 10 min. The UV-Vis spectra of the supernatants are presented in Figure 2b. They clearly showed a drastic decrease of SPR peak intensity of AuNPs after immobilization using APTES deposited zeolite, while the SPR peak was still observed after immobilization using MPTES deposited zeolite. These results clearly demonstrated that the APTES are better anchoring molecules for gold immobilization.

**Figure 3:**
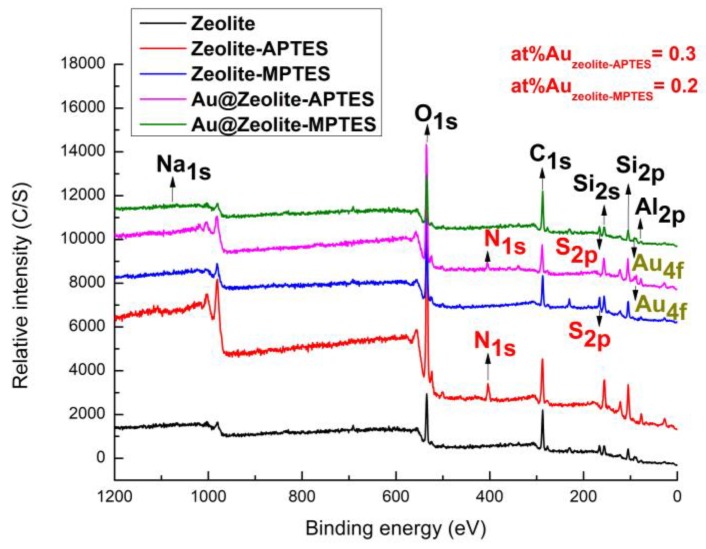
XPS survey scan spectra of zeolite, APTES/MPTES deposited zeolite and AuNPs immobilized zeolite.

All the samples were further characterized by XPS analysis and the XPS spectra are presented in Figure 3. After silane deposition, the intensity of the peaks corresponding to O, Al and Si elements showed an obvious increase as compared with the original zeolite. A new characteristic peak present at 400/162 eV could respectively correspond to the presence of the N_1s_/S_2p_ peak. Moreover, the content of nitrogen and sulfur were calculated from the XPS survey scan spectrum: at%N_1s_ = 6.9, at%S_2p_ = 8.8. The results indicated that a pretty good deposition of APTES/MPTES has been accomplished [36,37]. Furthermore, a good gold attachment on both surfaces was confirmed, as the Au_4f_ peaks at about 84 eV were observed in the survey scan spectra of AuNPs immobilized samples [38]. The AuNPs loading amount could also be seen from the XPS survey scan spectrum and a higher gold amount was obtained from Au@zeolite-APTES (at%Au_zeolite-APTES_ = 0.3 vs. at%Au_zeolite-MPTES_ = 0.2). Thus, the higher mole ratio calculated from XPS results (Au/APTES = 0.043 vs. Au/MPTES = 0.023) indicated that less APTES are required compared to MPTES to immobilize one AuNP.

**Figure 4:**
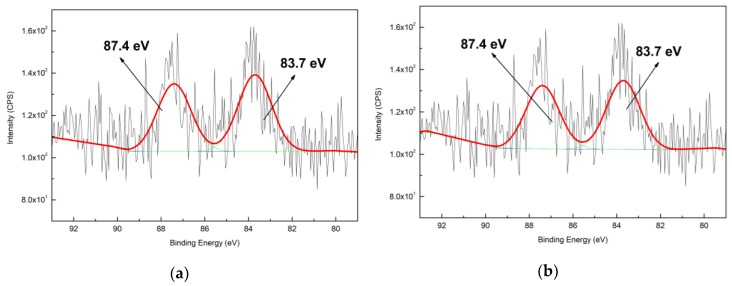
High resolution Au4f spectra of AuNPs immobilized zeolite: (**a**) Au@zeolite-APTES; (**b**) Au@zeolite-MPTES.

The high resolution Au_4f_ XPS spectra of Au@zeolite-APTES and Au@zeolite-MPTES are presented in Figure 4. The deconvolution of the spectra showed Au_4f7/2_ and Au_4f5/2_ components at 83.7 and 87.4 eV, respectively. According to literature [39], it might be attributed to Au (0) specie. However, compared with the BE value of bulk metallic gold of 84.0 and 88.0 eV [40], a shift of 0.3–0.6 eV occurred in the present work. The slight shift toward lower value could be attributed to the small size and negative charging of AuNPs. As the work function of Au = 5.27 eV is relatively high, the AuNPs probably obtain electron from Al (4.28 eV) and Si (4.85 eV) in the frame of Y zeolite [41]. Both gold immobilized samples revealed the presence of metallic gold species.

**Figure 5:**
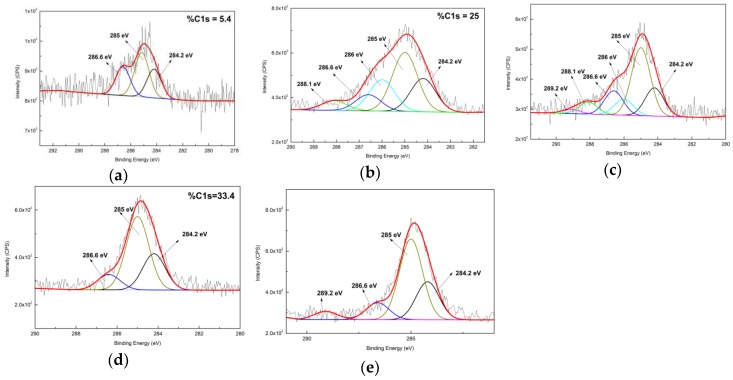
High resolution C_1s_ spectra of zeolite, silane deposited zeolite and AuNPs immobilized zeolite: (**a**) untreated zeolite; (**b**) APTES deposite zeolite; (**c**) Au@zeolite-APTES; (**d**) MPTES deposited zeolite; (**e**) Au@zeolite-MPTES.

**Figure 6:**
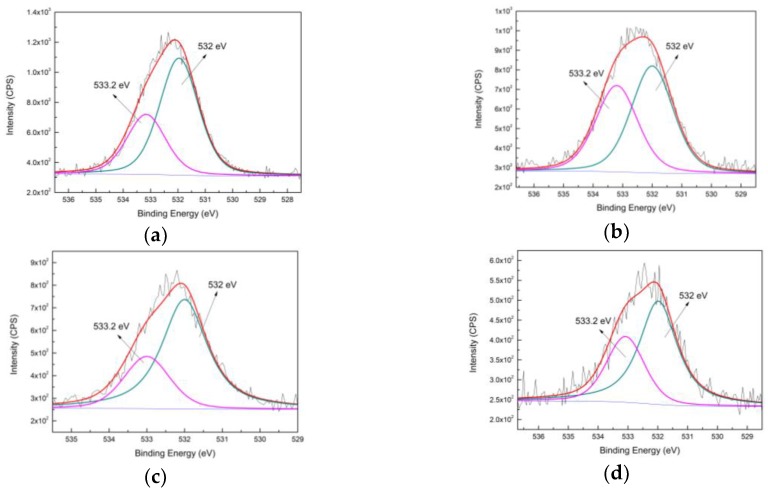
High resolution O_1s_ spectra of silane deposited zeolite and AuNPs immobilized zeolite: (**a**) APTES deposited zeolite; (**b**) Au@zeolite-APTES; (**c**) MPTES deposited zeolite; (**d**) Au@zeolite-MPTES.

Figure 5 displays the C_1s_ XPS spectra of original zeolite, silane deposited zeolite and AuNPs immobilized zeolite. A relative low amount of carbon was detected from the original zeolite (about %C_1s_ = 5.4), and three peaks were subsequently deconvoluted in Figure 6a: a main peak at 285 eV relating to C–C/C–H; a lower BE peak at 284.2 eV relating to C–Si; and a high BE peak at 286.6 eV relating to C–O. The presence of carbon is probably due to the adsorption of CO_2_ and/or existence of residual carbonaceous materials used during the synthesis of the zeolite [33,42]. An increase of carbon amount to %C_1s_ = 25 was observed in the spectrum of APTES treated zeolite in Figure 5b, evidencing a good coverage of ATPES molecules on the zeolite surface. Five peaks were deconvoluted in the C_1s_ spectrum: a main peak at 285 eV corresponding to methylene carbons; a low BE peak at 284.2 eV corresponding to C–Si from silane; three high BE peaks at 286 eV, 286.6 eV and 288.1 eV, corresponding to C–N from amine, C–O from ethoxy and C=O from amide, agreeing with the as-reported results [43].

The increase of the carbon amount (%C_1s_ = 33.4) was also seen from the spectrum of MPTES deposited zeolite in Figure 5d. The C_1s_ spectrum could be deconvoluted into three peaks: a main peak at 285 eV due to methylene carbon and carbon bonding sulfur [44,45,46]; a low BE peak at 284.2 eV according to C–Si from silane; and a high BE peak at 286.6 eV as a result of carbon bonding oxygen [47]. The C_1s_ spectra of AuNPs immobilized zeolite were respectively deconvoluted into the peaks that were previously mentioned in Figure 5c,e. Interestingly, a new peak at 289.2 eV according to O–C=O was observed in both spectra, and could probably represent the citrate anions of AuNPs, confirming that the AuNPs have been successfully immobilized on both surfaces.

As shown in Figure 6, the O_1s_ spectrum could be deconvoluted into two peaks: a peak at low binding energy of 532 eV assigned to O–Si; and another peak at high binding energy of 533.2 eV corresponding to O–C and/or O=C [48,49]. It was seen that the contribution of the peak at 533.2 eV increased after immobilization in both spectra that used ATPES and MPTES. Concerning the citrate existing on AuNPs, the increase of O–C/O=C bonds is probably due to the successful anchoring of gold on zeolite-APTES and zeolite-MPTES surface.

**Figure 7:**
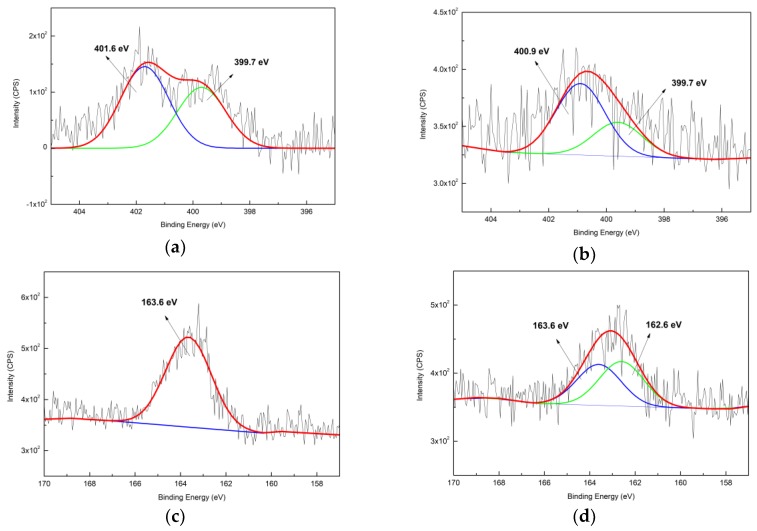
High resolution N_1s_ spectra and S_2p_ spectra of silane deposited zeolite and AuNPs immobilized zeolite: (**a**) N_1s_ of APTES deposited zeolite; (**b**) N_1s_ of Au@zeolite-APTES; (**c**) S_2p_ of MPTES deposited zeolite; (**d**) S_2p_ of Au@zeolite-MPTES.

The N_1s_ XPS spectra of zeolite-APTES and Au@zeolite-APTES samples are respectively shown in Figure 7a,b. Two peaks were seen in the spectrum of zeolite-APTES: a peak at 399.7 eV corresponding to amide or amine functions from APTES; a peak at 401.6 eV corresponding to -NH_3_^+^ from protonated amines [50,51]. After the immobilization of AuNPs, a decrease of BE peak at 399.7 eV was observed. Meanwhile, the higher BE peak was seen to shift to 400.9 eV, which is probably due to protonated amines binding with AuNPs. The contribution of high BE peak also increased after immobilization, implying more amines are well protonated in the suspension of pH = 4.2. Furthermore, the high BE peak at 401.6 eV shifted to a low BE position of 400.9 eV. The slight shift of 0.7 eV is probably due to the attachment between -NH_3_^+^ and Au [52]. All the results evidenced that the pH value of the AuNPs suspension at 4.2 could promote protonation of the amine groups (pKa = 9) resulting in a successful anchoring of the AuNPs.

The S_2p_ XPS spectra of zeolite-MPTES and Au@zeolite-MPTES samples are presented in Figure 7c,d, respectively. After MPTES deposition, a sole peak at 163.6 eV corresponding to S_2p3/2_ was observed, indicating the presence of reduced sulfur [28]. After immobilizing AuNPs, another new peak was seen at 162.6 eV. Based on as-reported results [47], it could be due to the gold-thiolate binding energy, as a result of the absorption of thiols on the gold surface. All the results confirmed the formation of thiol-gold bond and the immobilization of AuNPs with thiol groups.

**Figure 8:**
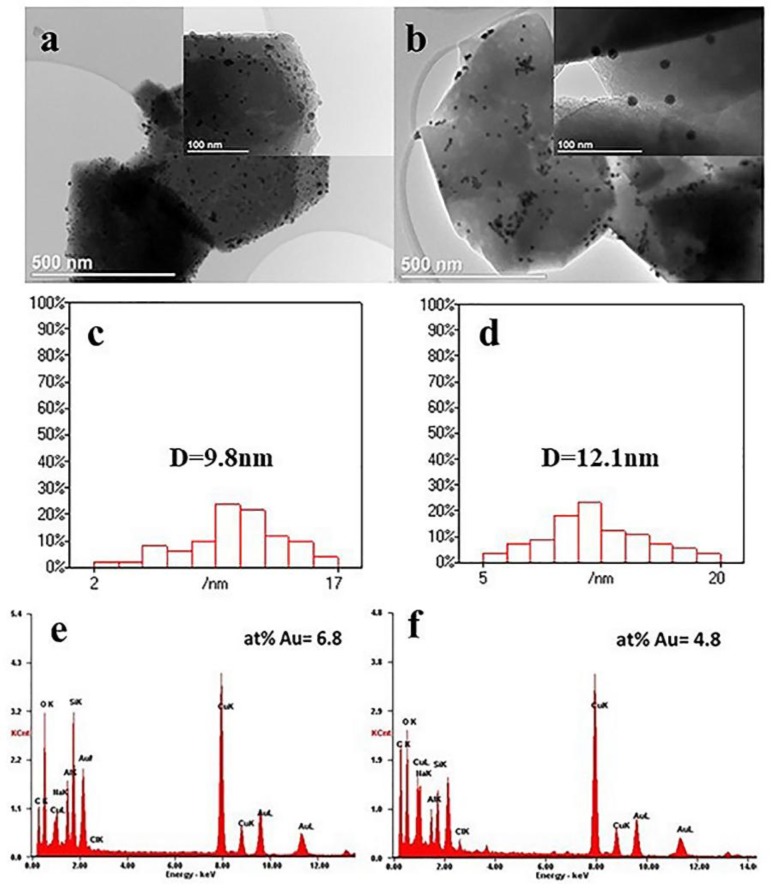
TEM images and EDS results of AuNPs immobilized zeolite: (**a**) TEM of Au@zeolite-APTES; (**b**) TEM of Au@zeolite-MPTES; (**c**) gold size distribution of Au@zeolite-APTES; (**d**) gold size distribution of Au@zeolite-MPTES; (**e**) EDS spectrum of Au@zeolite-APTES; (**f**) EDS spectrum of Au@zeolite-MPTES.

To further estimate the amount of AuNPs on the surfaces of the zeolite after immobilization, the samples were investigated using TEM and EDS. As presented in Figure 8, the dark spots representing spherical AuNPs were clearly observed on APTES and MPTES treated zeolite surfaces, showing a range of particle size of 10~15 nm (Figure 8c,d), in accordance with UV-Vis results. In comparison, the surfaces of APTES deposited zeolite exhibited a denser dispersion of AuNPs (Figure 8a vs. Figure 8b). Furthermore, a higher amount of AuNPs (6.8% vs. 4.8%) was also observed from the EDS results in Figure 8e,f. These results are in good agreement with the results obtained by UV-Vis and XPS.

**Figure 9:**
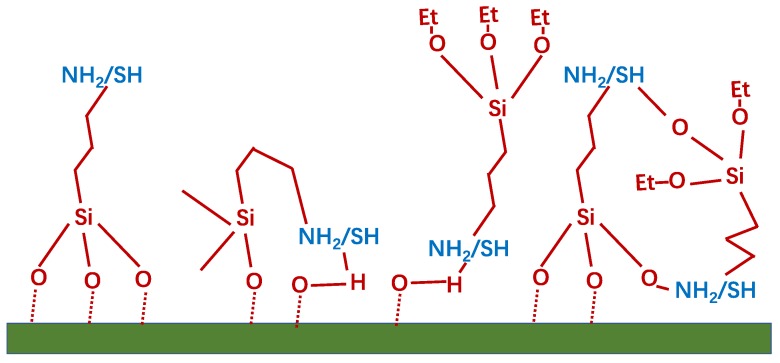
Schematic representation of possible structures on surface after APTES/MPTES deposition.

As reported by other groups [29,53], thiol functional groups could provide a covalent link to AuNPs, which has been announced to have a better ability than amine functional groups for attaching AuNPs. On the contrary, the amines groups of APTES provide higher gold loading amount on the zeolite in our study. Based on the XPS results, at%N_1s_ = 6.9 and at%S_2p_ = 8.8 were obtained from APTES and MPTES functionalized zeolite. As each APTES or MPTES molecular only owns a N or S, it seems likely that more thiols were deposited on the surface. As a result, there should be more AuNPs anchored on the MPTES deposited zeolite surface. However, it has been highlighted in many reports that organosilanes—as used herein for surface functionalization—are able to polymerize, especially in presence of water, forming a number of possible 2D and 3D surface structures [54,55,56,57]. As shown in Figure 9, APTES/MPTES molecules deposited on the surface could enable the appearance of various surface structures with different number of active functions for immobilization. Hence it means that not every amine or thiol group deposited on the surface is active for anchoring AuNPs. Thus, the less AuNPs immobilized on MPTES deposited zeolite could be due to less active functions existing on the as-functionalized surface for further immobilizing AuNPs. On the other hand, it is known that sulfur derivatives, such as thiols, are one of the most effective components enabling for the formation of self-assembled monolayers (SAMs) on gold. Aliphatic thiols, in particular, are considered as the most powerful agents in SAMs formation [58,59]. The most important difference between the as-reported results and the results in the present study are: (1) the difference in the structure of a metal oxide and a zeolite; and (2) in this study, our approach is based on a self-assembly approach, where preformed gold NPs attached to the surface of the zeolite, while others formed the gold NPs by impregnation of the support with the gold salt hydrate Au (3+) followed by its reduction to Au (0) [60]. Therefore, thiols are also able to form SAMs on the surface of gold during the AuNPs immobilization process in our work. Compared with amine, more thiols are preferred to attach onto the surface of a single gold nanoparticle, which decreases the number of immobilized AuNPs in accordance with the molar ratio obtained from XPS.

## 4. Conclusions

In this work, we studied the potentiality of APTES and MPTES to functionalize zeolite surface and the ability of amine/thiol attached surfaces further immobilizing gold nanoparticles. A successful deposition of APTES or MPTES on the zeolite surface was confirmed using Zeta potential, DRIFT and XPS characterizations. Spherical AuNPs were subsequently immobilized upon these as-deposited layers. TEM images revealed a higher density of gold nanoparticles covering APTES deposited zeolite. Moreover, EDS results showed a higher amount of AuNPs (at%_APTES_ = 6.8 vs. at%_MPTES_ = 4.8), which is in agreement with UV-Vis and XPS characterizations. The results suggested that APTES with amine showed better AuNPs immobilization ability than MPTES with thiol. The higher gold loading could be attributed to as follows: on one hand, the less active functions for immobilization were obtained during functionalization; on the other hand, thiols with the powerful ability to form SAMs were preferred to attach to the surface of a single preformed gold nanoparticle, resulting in the decrease of immobilized AuNPs.

## Figures and Tables

**Table 1:** Zeta potential of original zeolite, silane modified zeolite and AuNPs immobilized zeolite.

Linkage Reagent	Original Zeolite (mV)	Silane Deposited Zeolite (mV)	AuNPs Immobilized Zeolite (mV)
APTES	−39.7 ± 2.6	−36.4 ± 1.6	−40.2 ± 2.5
MPTES	−39.7 ± 1.8	−12.2 ± 1.1	−44.2 ± 2.4
